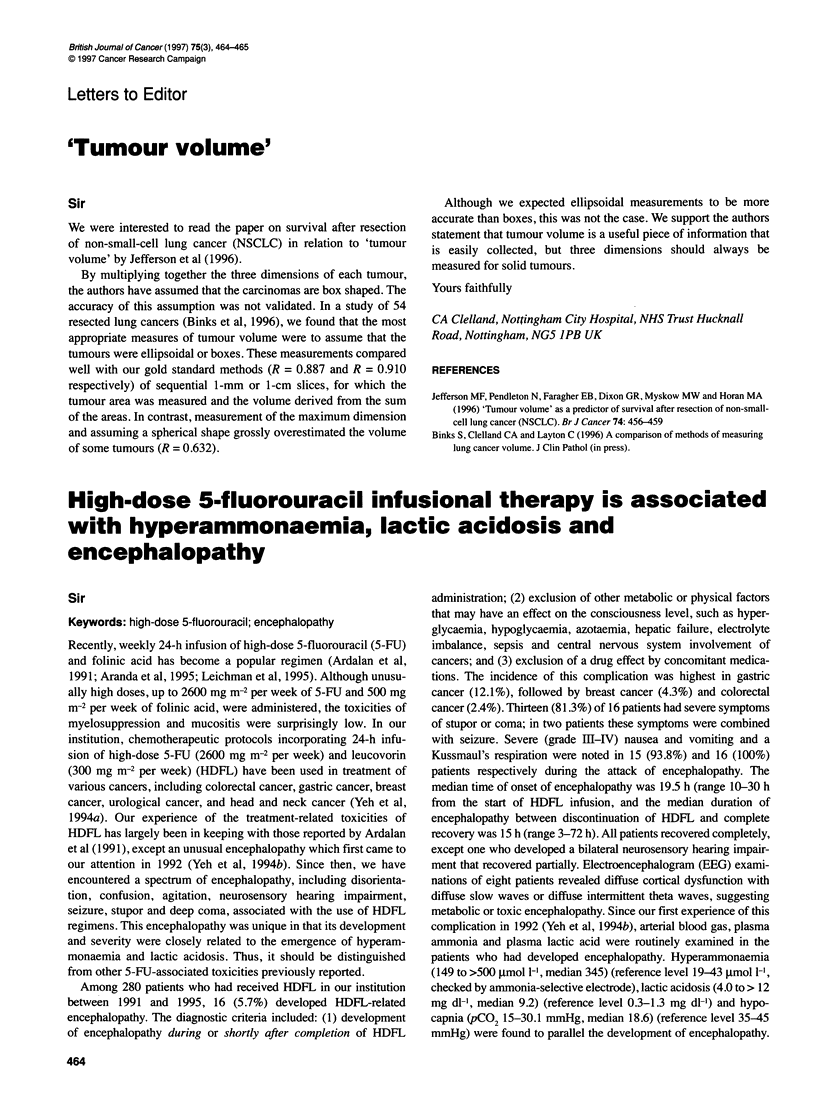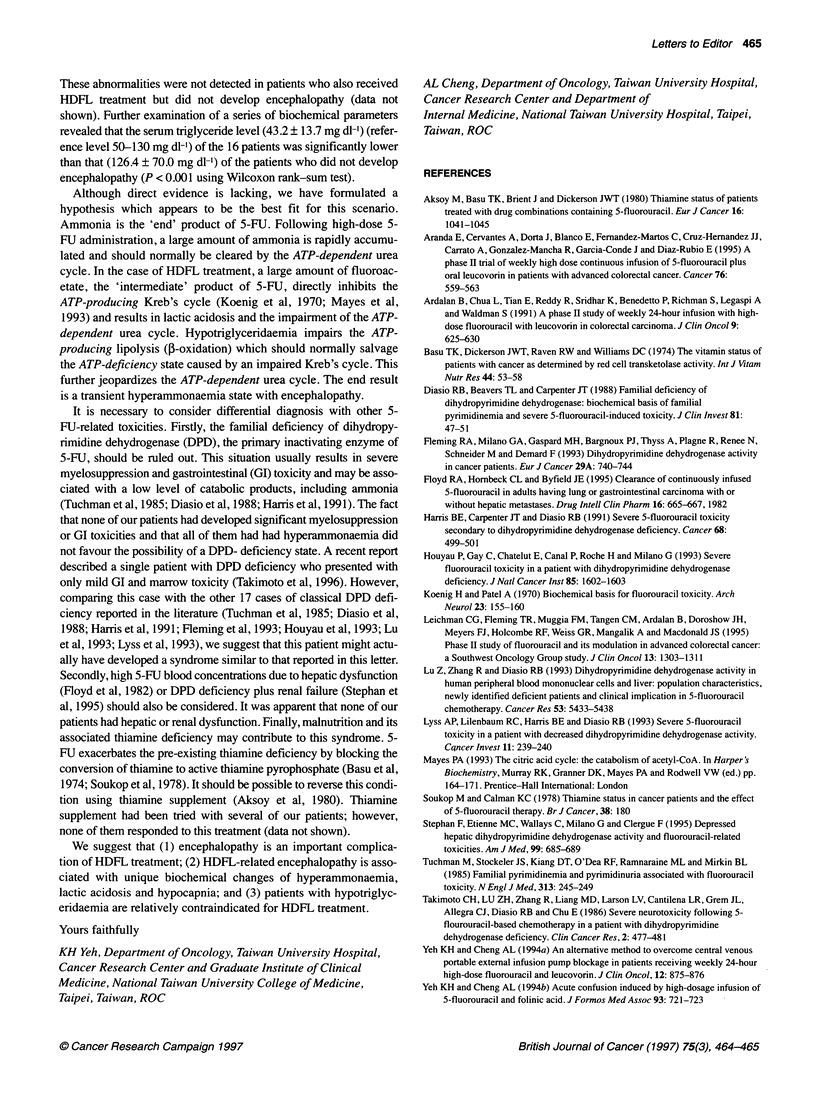# High-dose 5-fluorouracil infusional therapy is associated with hyperammonaemia, lactic acidosis and encephalopathy.

**DOI:** 10.1038/bjc.1997.79

**Published:** 1997

**Authors:** K. H. Yeh, A. L. Cheng


British Journal of Cancer (1997) 75(3), 464–465

© 1997 Cancer Research Campaign

Letters to Editor

### High-dose 5-fluorouracil infusional therapy is associated with hyperammonaemia, lactic acidosis and encephalopathy

### Sir

**Keywords:** high-dose 5-fluorouracil; encephalopathy

Recently, weekly 24-h infusion of high-dose 5-fluorouracil (5-FU) and folinic acid has become a popular regimen (Ardalan et al, 1991; Aranda et al, 1995; Leichman et al, 1995). Although unusually high doses, up to 2600 mg $m^{-2}$ per week of 5-FU and 500 mg $m^{-2}$ per week of folinic acid, were administered, the toxicities of myelosuppression and mucositis were surprisingly low. In our institution, chemotherapeutic protocols incorporating 24-h infusion of high-dose 5-FU (2600 mg $m^{-2}$ per week) and leucovorin (300 mg $m^{-2}$ per week) (HDFL) have been used in treatment of various cancers, including colorectal cancer, gastric cancer, breast cancer, urological cancer, and head and neck cancer (Yeh et al, 1994*a*). Our experience of the treatment-related toxicities of HDFL has largely been in keeping with those reported by Ardalan et al (1991), except an unusual encephalopathy which first came to our attention in 1992 (Yeh et al, 1994*b*). Since then, we have encountered a spectrum of encephalopathy, including disorientation, confusion, agitation, neurosensory hearing impairment, seizure, stupor and deep coma, associated with the use of HDFL regimens. This encephalopathy was unique in that its development and severity were closely related to the emergence of hyperammonaemia and lactic acidosis. Thus, it should be distinguished from other 5-FU-associated toxicities previously reported.

Among 280 patients who had received HDFL in our institution between 1991 and 1995, 16 (5.7%) developed HDFL-related encephalopathy. The diagnostic criteria included: (1) development of encephalopathy *during* or *shortly after completion* of HDFL administration; (2) exclusion of other metabolic or physical factors that may have an effect on the consciousness level, such as hyperglycaemia, hypoglycaemia, azotaemia, hepatic failure, electrolyte imbalance, sepsis and central nervous system involvement of cancers; and (3) exclusion of a drug effect by concomitant medications. The incidence of this complication was highest in gastric cancer (12.1%), followed by breast cancer (4.3%) and colorectal cancer (2.4%). Thirteen (81.3%) of 16 patients had severe symptoms of stupor or coma; in two patients these symptoms were combined with seizure. Severe (grade III–IV) nausea and vomiting and a Kussmaul's respiration were noted in 15 (93.8%) and 16 (100%) patients respectively during the attack of encephalopathy. The median time of onset of encephalopathy was 19.5 h (range 10–30 h from the start of HDFL infusion, and the median duration of encephalopathy between discontinuation of HDFL and complete recovery was 15 h (range 3–72 h). All patients recovered completely, except one who developed a bilateral neurosensory hearing impairment that recovered partially. Electroencephalogram (EEG) examinations of eight patients revealed diffuse cortical dysfunction with diffuse slow waves or diffuse intermittent theta waves, suggesting metabolic or toxic encephalopathy. Since our first experience of this complication in 1992 (Yeh et al, 1994*b*), arterial blood gas, plasma ammonia and plasma lactic acid were routinely examined in the patients who had developed encephalopathy. Hyperammonaemia (149 to >500 μmol $l^{-1}$, median 345) (reference level 19–43 μmol $l^{-1}$, checked by ammonia-selective electrode), lactic acidosis (4.0 to > 12 mg $dl^{-1}$, median 9.2) (reference level 0.3–1.3 mg $dl^{-1}$) and hypocapnia ($pCO_2$ 15–30.1 mmHg, median 18.6) (reference level 35–45 mmHg) were found to parallel the development of encephalopathy.

These abnormalities were not detected in patients who also received HDFL treatment but did not develop encephalopathy (data not shown). Further examination of a series of biochemical parameters revealed that the serum triglyceride level ($43.2 \pm 13.7$ mg $dl^{-1}$) (reference level 50–130 mg $dl^{-1}$) of the 16 patients was significantly lower than that ($126.4 \pm 70.0$ mg $dl^{-1}$) of the patients who did not develop encephalopathy ($P < 0.001$ using Wilcoxon rank–sum test).

Although direct evidence is lacking, we have formulated a hypothesis which appears to be the best fit for this scenario. Ammonia is the 'end' product of 5-FU. Following high-dose 5-FU administration, a large amount of ammonia is rapidly accumulated and should normally be cleared by the *ATP-dependent* urea cycle. In the case of HDFL treatment, a large amount of fluoroacetate, the 'intermediate' product of 5-FU, directly inhibits the *ATP-producing* Kreb's cycle (Koenig et al, 1970; Mayes et al, 1993) and results in lactic acidosis and the impairment of the *ATP-dependent* urea cycle. Hypotriglyceridaemia impairs the *ATP-producing* lipolysis (β-oxidation) which should normally salvage the *ATP-deficiency* state caused by an impaired Kreb's cycle. This further jeopardizes the *ATP-dependent* urea cycle. The end result is a transient hyperammonaemia state with encephalopathy.

It is necessary to consider differential diagnosis with other 5-FU-related toxicities. Firstly, the familial deficiency of dihydropyrimidine dehydrogenase (DPD), the primary inactivating enzyme of 5-FU, should be ruled out. This situation usually results in severe myelosuppression and gastrointestinal (GI) toxicity and may be associated with a low level of catabolic products, including ammonia (Tuchman et al, 1985; Diasio et al, 1988; Harris et al, 1991). The fact that none of our patients had developed significant myelosuppression or GI toxicities and that all of them had had hyperammonaemia did not favour the possibility of a DPD- deficiency state. A recent report described a single patient with DPD deficiency who presented with only mild GI and marrow toxicity (Takimoto et al, 1996). However, comparing this case with the other 17 cases of classical DPD deficiency reported in the literature (Tuchman et al, 1985; Diasio et al, 1988; Harris et al, 1991; Fleming et al, 1993; Houyau et al, 1993; Lu et al, 1993; Lyss et al, 1993), we suggest that this patient might actually have developed a syndrome similar to that reported in this letter. Secondly, high 5-FU blood concentrations due to hepatic dysfunction (Floyd et al, 1982) or DPD deficiency plus renal failure (Stephan et al, 1995) should also be considered. It was apparent that none of our patients had hepatic or renal dysfunction. Finally, malnutrition and its associated thiamine deficiency may contribute to this syndrome. 5-FU exacerbates the pre-existing thiamine deficiency by blocking the conversion of thiamine to active thiamine pyrophosphate (Basu et al, 1974; Soukop et al, 1978). It should be possible to reverse this condition using thiamine supplement (Aksoy et al, 1980). Thiamine supplement had been tried with several of our patients; however, none of them responded to this treatment (data not shown).

We suggest that (1) encephalopathy is an important complication of HDFL treatment; (2) HDFL-related encephalopathy is associated with unique biochemical changes of hyperammonaemia, lactic acidosis and hypocapnia; and (3) patients with hypotriglyceridaemia are relatively contraindicated for HDFL treatment.

Yours faithfully

*KH Yeh, Department of Oncology, Taiwan University Hospital, Cancer Research Center and Graduate Institute of Clinical Medicine, National Taiwan University College of Medicine, Taipei, Taiwan, ROC*

*AL Cheng, Department of Oncology, Taiwan University Hospital, Cancer Research Center and Department of Internal Medicine, National Taiwan University Hospital, Taipei, Taiwan, ROC*

### REFERENCES

Aksoy M, Basu TK, Brient J and Dickerson JWT (1980) Thiamine status of patients treated with drug combinations containing 5-fluorouracil. *Eur J Cancer* **16**: 1041–1045

Aranda E, Cervantes A, Dorta J, Blanco E, Fernandez-Martos C, Cruz-Hernandez JJ, Carrato A, Gonzalez-Mancha R, Garcia-Conde J and Diaz-Rubio E (1995) A phase II trial of weekly high dose continuous infusion of 5-fluorouracil plus oral leucovorin in patients with advanced colorectal cancer. *Cancer* **76**: 559–563

Ardalan B, Chua L, Tian E, Reddy R, Sridhar K, Benedetto P, Richman S, Legaspi A and Waldman S (1991) A phase II study of weekly 24-hour infusion with high-dose fluorouracil with leucovorin in colorectal carcinoma. *J Clin Oncol* **9**: 625–630

Basu TK, Dickerson JWT, Raven RW and Williams DC (1974) The vitamin status of patients with cancer as determined by red cell transketolase activity. *Int J Vitam Nutr Res* **44**: 53–58

Diasio RB, Beavers TL and Carpenter JT (1988) Familial deficiency of dihydropyrimidine dehydrogenase: biochemical basis of familial pyrimidinemia and severe 5-fluorouracil-induced toxicity. *J Clin Invest* **81**: 47–51

Fleming RA, Milano GA, Gaspard MH, Bargnoux PJ, Thyss A, Plagne R, Renee N, Schneider M and Demard F (1993) Dihydropyrimidine dehydrogenase activity in cancer patients. *Eur J Cancer* **29A**: 740–744

Floyd RA, Hornbeck CL and Byfield JE (1995) Clearance of continuously infused 5-fluorouracil in adults having lung or gastrointestinal carcinoma with or without hepatic metastases. *Drug Intell Clin Pharm* **16**: 665–667, 1982

Harris BE, Carpenter JT and Diasio RB (1991) Severe 5-fluorouracil toxicity secondary to dihydropyrimidine dehydrogenase deficiency. *Cancer* **68**: 499–501

Houyau P, Gay C, Chatelut E, Canal P, Roche H and Milano G (1993) Severe fluorouracil toxicity in a patient with dihydropyrimidine dehydrogenase deficiency. *J Natl Cancer Inst* **85**: 1602–1603

Koenig H and Patel A (1970) Biochemical basis for fluorouracil toxicity. *Arch Neurol* **23**: 155–160

Leichman CG, Fleming TR, Muggia FM, Tangen CM, Ardalan B, Doroshow JH, Meyers FJ, Holcombe RF, Weiss GR, Mangalik A and Macdonald JS (1995) Phase II study of fluorouracil and its modulation in advanced colorectal cancer: a Southwest Oncology Group study. *J Clin Oncol* **13**: 1303–1311

Lu Z, Zhang R and Diasio RB (1993) Dihydropyrimidine dehydrogenase activity in human peripheral blood mononuclear cells and liver: population characteristics, newly identified deficient patients and clinical implication in 5-fluorouracil chemotherapy. *Cancer Res* **53**: 5433–5438

Lyss AP, Lilenbaum RC, Harris BE and Diasio RB (1993) Severe 5-fluorouracil toxicity in a patient with decreased dihydropyrimidine dehydrogenase activity. *Cancer Invest* **11**: 239–240

Mayes PA (1993) The citric acid cycle: the catabolism of acetyl-CoA. In *Harper's Biochemistry*, Murray RK, Granner DK, Mayes PA and Rodwell VW (ed.) pp. 164–171. Prentice–Hall International: London

Soukop M and Calman KC (1978) Thiamine status in cancer patients and the effect of 5-fluorouracil therapy. *Br J Cancer*, **38**: 180

Stephan F, Etienne MC, Wallays C, Milano G and Clergue F (1995) Depressed hepatic dihydropyrimidine dehydrogenase activity and fluorouracil-related toxicities. *Am J Med*, **99**: 685–689

Tuchman M, Stockeler JS, Kiang DT, O'Dea RF, Ramnaraine ML and Mirkin BL (1985) Familial pyrimidinemia and pyrimidinuria associated with fluorouracil toxicity. *N Engl J Med*, **313**: 245–249

Takimoto CH, LU ZH, Zhang R, Liang MD, Larson LV, Cantilena LR, Grem JL, Allegra CJ, Diasio RB and Chu E (1986) Severe neurotoxicity following 5-flourouracil-based chemotherapy in a patient with dihydropyrimidine dehydrogenase deficiency. *Clin Cancer Res*, **2**: 477–481

Yeh KH and Cheng AL (1994*a*) An alternative method to overcome central venous portable external infusion pump blockage in patients receiving weekly 24-hour high-dose fluorouracil and leucovorin. *J Clin Oncol*, **12**: 875–876

Yeh KH and Cheng AL (1994*b*) Acute confusion induced by high-dosage infusion of 5-fluorouracil and folinic acid. *J Formos Med Assoc* **93**: 721–723

© Cancer Research Campaign 1997